# Association Between Obstructive Sleep Apnea and Cardiovascular Risk: A Systematic Review and Meta-Analysis of Prospective Cohort Studies

**DOI:** 10.3390/medicina61111988

**Published:** 2025-11-05

**Authors:** Maria-Laura Craciun, Adina-Cristiana Avram, Florina Buleu, Marius Badalica-Petrescu, Ioana-Georgiana Cotet, Diana-Maria Mateescu, Stela Iurciuc, Simina Crisan, Ana-Olivia Toma, Claudiu Avram, Ana-Maria Pah

**Affiliations:** 1 Cardiology Department, “Victor Babes” University of Medicine and Pharmacy, Eftimie Murgu Square 2, 300041 Timisoara, Romania; laura.craciun@umft.ro (M.-L.C.); iurciuc.stela@umft.ro (S.I.); anamaria.pah@umft.ro (A.-M.P.); 2 Department of Internal Medicine I, “Victor Babes” University of Medicine and Pharmacy, Eftimie Murgu Square 2, 300041 Timisoara, Romania; 3 Department VI, Discipline of Internal Medicine and Ambulatory Care, Prevention and Cardiovascular Recovery, Faculty of Medicine, “Victor Babes” University of Medicine and Pharmacy Timisoara, E. Murgu Square, No. 2, 300041 Timisoara, Romania; 4 Doctoral School, Department of General Medicine, “Victor Babes” University of Medicine and Pharmacy, Eftimie Murgu Square 2, 300041 Timisoara, Romania; 5 Center for the Morphologic Study of the Skin (MORPHODERM), “Victor Babes” University of Medicine and Pharmacy Timisoara, 300041 Timisoara, Romania; 6 Department XVI-Balneology, Medical Recovery and Rheumatology, “Victor Babes” University of Medicine and Pharmacy, 300041 Timisoara, Romania; avram.claudiu@umft.ro

**Keywords:** obstructive sleep apnea, cardiovascular risk, meta-analysis, cohort studies, continuous positive airway pressure, hazard ratio, CPAP adherence, dose–response

## Abstract

*Background and Objectives*: Obstructive sleep apnea (OSA) is a prevalent disorder associated with increased cardiovascular (CV) risk. *Materials and Methods*: We conducted a PRISMA-compliant systematic review and meta-analysis of prospective cohort studies assessing OSA and incident CV outcomes. *Results*: From 2463 records, 18 studies (>25,000 participants; median follow-up 9 years) were included. OSA was associated with increased CV risk (pooled HR 1.82, 95% CI 1.45–2.28). Dose–response analysis showed a progressive risk increase: mild OSA (HR 1.21, 95% CI 0.98–1.50), moderate (HR 1.56, 95% CI 1.20–2.03), and severe (HR 2.45, 95% CI 1.85–3.25). Continuous positive airway pressure (CPAP) adherence (≥4 h/night) reduced risk (HR 0.76, 95% CI 0.60–0.96). *Conclusions*: OSA confers a severity-dependent CV risk, which is mitigated by adequate CPAP adherence. Systematic screening and adherence support may reduce CV morbidity and mortality. PROSPERO: CRD420251168363.

## 1. Introduction

Obstructive sleep apnea (OSA) is a common sleep-related breathing disorder characterized by recurrent episodes of partial or complete upper airway obstruction during sleep, leading to intermittent hypoxemia and sleep fragmentation. The severity of OSA is quantified using the apnea–hypopnea index (AHI), defined as the number of apneas and hypopneas per hour of sleep—mild (≥5/h), moderate (≥15/h), and severe (≥30/h) [1]. These definitions and thresholds are standardized by the American Academy of Sleep Medicine (AASM) scoring manual, including the 2017 updates.

The prevalence of OSA varies widely according to age, sex, and obesity. A systematic review estimated that 6–17% of adults have moderate-to-severe OSA (AHI ≥ 15), with higher rates observed in older populations [2]. Population-based cohorts have shown substantial increases in prevalence over recent decades [3]. In the HypnoLaus community study, moderate-to-severe OSA affected approximately 23% of women and 50% of men [4]. A global analysis projected that hundreds of millions of adults are affected worldwide, with significant geographic variability and a major burden on public health [5]. Despite this high prevalence, OSA remains markedly underdiagnosed in the general population [2].

The link between OSA and cardiovascular (CV) disease is biologically plausible and multifactorial. Repeated cycles of hypoxia and reoxygenation induce oxidative stress, systemic inflammation, and endothelial dysfunction—key mechanisms in atherogenesis [6,7,8]. Chronic sympathetic activation contributes to sustained hypertension and increased blood pressure variability [9]. These pathophysiological cascades also promote insulin resistance, dyslipidemia, hypercoagulability, and arterial stiffness, fostering coronary and cerebrovascular events [6,10,11,12]. The 2021 American Heart Association scientific statement highlights OSA as an independent risk factor for cardiovascular morbidity and mortality through these pathways [12]. Furthermore, therapeutic interventions like continuous positive airway pressure (CPAP) and mandibular advancement devices (MAD) have demonstrated comparable efficacy in reducing blood pressure in OSA patients, underscoring their role in addressing sympathetic overactivation and hypertension [13]. Unlike prior meta-analyses, our study integrates data through 2025, incorporates E-value sensitivity analysis to quantify unmeasured confounding, and performs a CPAP adherence-based subanalysis. These features enhance causal inference and clinical applicability.

Given the growing global burden of OSA and the established biological rationale linking it to cardiovascular disease, this systematic review and meta-analysis aim to quantitatively synthesize evidence from prospective cohort studies to assess the association between OSA and incident cardiovascular outcomes, including coronary heart disease, heart failure, and stroke, and to evaluate the potential modifying effect of OSA severity and continuous positive airway pressure (CPAP) therapy. Compared with previous meta-analyses that included mixed study designs or data only up to 2021, our study is the first to synthesize exclusively prospective cohort evidence through 2025, integrate E-value causal sensitivity analysis, and evaluate CPAP adherence as a modifier of cardiovascular outcomes.

## 2. Materials and Methods

### 2.1. Study Design and Reporting Framework

This systematic review and meta-analysis were conducted and reported in accordance with the Preferred Reporting Items for Systematic Reviews and Meta-Analyses (PRISMA) 2020 guidelines [14]. The study protocol was prospectively registered in the International Prospective Register of Systematic Reviews (PROSPERO) under the identifier CRD420251168363, registration date: 14 October 2025. All methodological steps adhered to the standards of the Cochrane Handbook for Systematic Reviews of Interventions [15].

### 2.2. PICO Framework and Research Question

The research question was structured according to the PICO (Population–Intervention–Comparator–Outcome) framework: Component: Adults (≥18 years) from population-based or clinical prospective cohort studies with baseline evaluation of OSA; Intervention/Exposure (I): Presence of obstructive sleep apnea (OSA), defined by the apnea–hypopnea index (AHI) ≥ 5 events/hour, and stratified by severity (mild, moderate, severe); Comparator (C): Participants without OSA (AHI < 5 events/hour); Outcomes (O): Incident cardiovascular outcomes, including coronary heart disease (CHD), heart failure (HF), stroke, and cardiovascular or all-cause mortality.

The primary objective was to quantitatively estimate the association between OSA and incident cardiovascular events; secondary aims included exploring associations by OSA severity and evaluating the potential modifying effect of continuous positive airway pressure (CPAP) therapy.

### 2.3. Eligibility Criteria

Inclusion criteria:

1. Design: Prospective or longitudinal cohort studies (community or clinical) with ≥12 months follow-up;
2. Exposure: OSA objectively diagnosed using PSG or home sleep apnea testing, classified by AHI thresholds;
3. Population: Adults (≥18 years) free from cardiovascular disease at baseline;
4. Outcomes: Incident cardiovascular events (CHD, HF, stroke, or mortality), reported as adjusted effect estimates (hazard ratio [HR], odds ratio [OR], or relative risk [RR]) with 95% confidence intervals (CIs);
5. Analysis: Multivariable adjustment for major confounders (age, sex, BMI, smoking, hypertension, diabetes);
6. Publication: Peer-reviewed, English-language original articles published between 2000–2025.

Exclusion criteria:

- Retrospective, case–control, or cross-sectional studies;
- Pediatric populations;
- Lack of objective OSA assessment;
- Reviews, editorials, conference abstracts;
- Duplicate cohorts. Population overlap across large cohorts (Sleep Heart Health Study, Wisconsin Sleep Cohort, Busselton Health Study) was carefully evaluated to avoid duplicate participant inclusion.

### 2.4. Search Strategy

A comprehensive and systematic search was conducted in PubMed/MEDLINE, Embase, Scopus, and Web of Science Core Collection databases, from inception to 30 September 2025, with an ad hoc verification on 16 October 2025 confirming no additional eligible prospective cohorts, in accordance with PRISMA 2020 recommendations. The search strategy combined controlled vocabulary (MeSH/Emtree terms) and free-text keywords as follows:

(“obstructive sleep apnea” OR “sleep-disordered breathing” OR “sleep apnea–hypopnea syndrome”) AND (“cardiovascular disease” OR “coronary heart disease” OR “myocardial infarction” OR “heart failure” OR “stroke” OR “mortality”) AND (“cohort” OR “prospective” OR “longitudinal”).

No date restrictions were imposed other than the final search date (30 September 2025).

The following filters were applied: humans, adults (≥18 years), English language, and original articles.

Search strings were adapted to the syntax of each database, and full strategies are provided in Supplementary Table S1. In addition, reference lists of all relevant reviews and eligible studies were manually screened to identify additional publications.

Two independent reviewers performed the database search, title/abstract screening, and full-text selection. Discrepancies were resolved by consensus or by consultation with a senior reviewer.

The search was last updated on 30 September 2025, and all retrieved records were managed using EndNote X20 (Clarivate Analytics) to remove duplicates prior to screening.

### 2.5. Study Screening and Eligibility Assessment

All retrieved records were imported into EndNote X20 (Clarivate Analytics) for automatic and manual duplicate removal. Study selection was conducted in two sequential stages, following PRISMA 2020 guidelines:

1. Title and abstract screening to exclude clearly irrelevant studies, non-original articles, case reports, editorials, or reviews.
2. Full-text assessment to evaluate eligibility against predefined inclusion and exclusion criteria (as detailed in Section 2.6).

Two reviewers independently screened all titles, abstracts, and full-texts. Discrepancies were resolved through discussion and consensus, and when necessary, adjudicated by a third senior reviewer.

**Figure 1:**
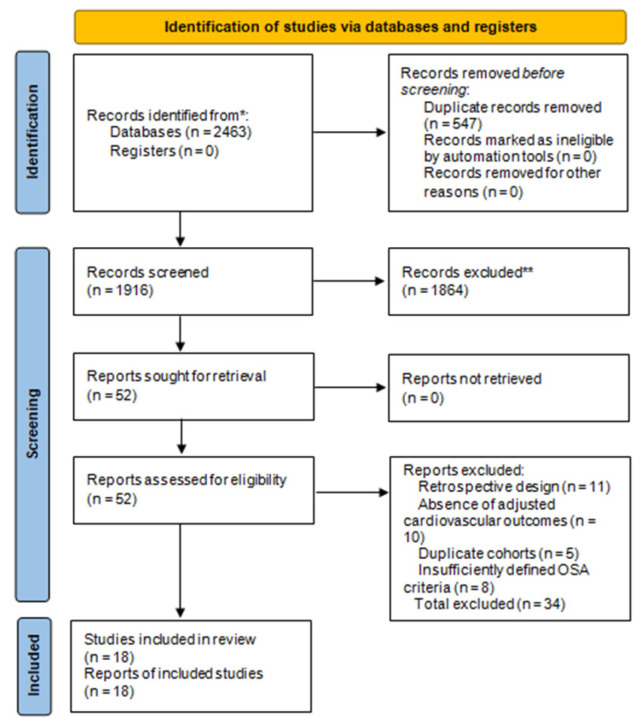
PRISMA 2020 Flow Diagram. Records identified (*n* = 2463), screened (*n* = 1916 after duplicates removed), full-text-assessed (*n* = 52), and included (*n* = 18). * Records identified from database searches conducted in PubMed/MEDLINE, Embase, Scopus, and Web of Science Core Collection (search date: 30 September 2025).

Inter-rater agreement for study inclusion was substantial (κ = 0.84), ensuring high reproducibility. The entire selection process was documented using a PRISMA 2020 flow diagram (Figure 1), which summarizes the number of records identified, screened, excluded, and finally included in the meta-analysis.

### 2.6. Data Extraction

Two reviewers independently extracted key data using a standardized Excel template: Study characteristics (author, year, country, cohort name, sample size, follow-up); Participant demographics (age, sex, BMI); OSA definitions and severity thresholds (AHI cutoffs); Outcomes assessed (CHD, HF, stroke, mortality); Covariates adjusted in the final models; Adjusted effect sizes (HR, OR, RR) and 95% CIs; Information on CPAP therapy (treated vs. untreated subgroups).

Discrepancies were resolved through consensus. Fully adjusted estimates were prioritized when multiple models were available. OSA was defined according to the American Academy of Sleep Medicine (AASM) criteria. Studies using pre-2012 definitions were analyzed separately in sensitivity tests to ensure comparability.

Cardiovascular outcomes were operationalized as investigator-defined or ICD-coded events, including coronary heart disease (CHD, ICD-10 I20–I25), heart failure (I50), stroke (I60–I69), and cardiovascular mortality (I00–I99).

### 2.7. Quality Assessment

The Newcastle–Ottawa Scale (NOS) was used to assess methodological quality [16]. This tool evaluates three domains: (1) Selection (representativeness, ascertainment, and baseline outcome absence); (2) Comparability (control for major confounders such as age, sex, BMI, and comorbidities); (3) Outcome (assessment method and adequacy of follow-up).

Scores ≥7 indicated high quality, 5–6 moderate, and <5 low quality. Sensitivity analyses excluding lower-quality studies were conducted to test robustness.

### 2.8. Statistical Analysis

All analyses were performed using Review Manager (RevMan, version 5.4; Cochrane Collaboration, Oxford, UK) and Comprehensive Meta-Analysis (CMA, version 4.0; Biostat, Englewood, NJ, USA). Effect sizes were expressed as hazard ratios (HRs) with 95% confidence intervals (CIs). When odds ratios (ORs) or risk ratios (RRs) were reported, they were converted to HRs using standard logarithmic transformations and variance propagation formulas [17].

A DerSimonian–Laird random-effects model was used to account for between-study heterogeneity (τ^2^), as clinical and methodological diversity across cohorts was anticipated. Heterogeneity was quantified using Cochran’s Q and I^2^ statistics, interpreted as low (<25%), moderate (25–75%), or high (>75%) [18].

In addition, 95% prediction intervals (PI) were calculated for all pooled estimates to indicate the range in which the true effect of a new study is expected to lie.

Subgroup analyses were predefined by: OSA severity (mild, moderate, severe); Type of cardiovascular outcome (CHD, HF, stroke, all-cause mortality); Sex (male vs. female); CPAP therapy status (treated vs. untreated).

Sensitivity analyses were conducted by (a) leave-one-out exclusion of each individual study, (b) restriction to high-quality studies (NOS ≥ 8), and (c) alternative variance estimators (REML) and Hartung–Knapp–Sidik–Jonkman adjustments, to verify robustness of pooled effects. Alternative τ^2^ estimators (REML, Paule–Mandel) produced identical pooled HRs to the third decimal, confirming robustness. Studies restricted to one sex (e.g., women-only cohorts) were included in the overall meta-analysis because they contributed to the estimation of sex-specific effects. Sex-stratified subgroup analyses were performed to assess consistency across sexes.

Publication bias was evaluated visually using funnel plots and statistically via Egger’s regression test and Begg’s rank-correlation test, both computed in CMA v4.0 [19].

E-values were calculated for the pooled HRs and for the lower limit of their 95% CIs to assess the minimum strength of unmeasured confounding required to nullify the observed association.

A two-tailed *p*-value < 0.05 was considered statistically significant. All analyses followed Cochrane and PRISMA 2020 recommendations for meta-analytical methodology. In addition, meta-regression explored potential sources of heterogeneity (e.g., mean cohort age, BMI). Meta-regression did not identify significant modifiers; coefficients and *p*-values for mean age (β = 0.015, *p* = 0.21), BMI (β = 0.023, *p* = 0.18), and sex proportion (β = −0.012, *p* = 0.27) are provided in Supplementary Table S2. Certainty of evidence was assessed using GRADE for observational studies, downgrading for risk of bias, inconsistency, indirectness, imprecision, and publication bias.

### 2.9. Ethics and Data Availability

This meta-analysis synthesized previously published, de-identified data and thus did not require ethical approval. All extracted data and analysis code are available upon reasonable request from the corresponding author.

## 3. Results

### 3.1. Study Selection

The systematic search retrieved 2463 records from PubMed, Embase, Scopus, and Web of Science. After removing 547 duplicates, 1916 unique titles were screened. Following title and abstract evaluation, 52 full-text articles were assessed for eligibility. Of these, 18 studies met all inclusion criteria and were included in the quantitative synthesis, as shown in Figure 1. Exclusions were primarily due to retrospective design (*n* = 11), absence of adjusted cardiovascular outcomes (*n* = 10), duplicate cohorts (*n* = 5), or insufficiently defined OSA criteria (*n* = 8).

The final dataset included 18 prospective cohort studies, comprising >25,000 participants, followed for 5–24 years, across North America, Europe, and Asia.

### 3.2. Study Characteristics

A total of 18 prospective cohort studies were included in the quantitative synthesis, as shown in Table 1. All studies assessed obstructive sleep apnea (OSA) using overnight polysomnography (PSG) or validated portable respiratory monitoring systems, with disease severity classified according to apnea–hypopnea index (AHI) thresholds: ≥5 events/hour for OSA diagnosis, ≥15 events/hour for moderate-to-severe OSA, and ≥30 events/hour for severe OSA.

Primary outcomes included incident stroke, coronary heart disease (CHD), heart failure (HF), cardiovascular (CV) mortality, and all-cause mortality. Follow-up duration across studies ranged from 4 to 20 years (median 9 years).

Most studies employed multivariable-adjusted Cox proportional hazards or logistic regression models, accounting for key confounders such as age, sex, body mass index (BMI), smoking status, hypertension, diabetes mellitus, and serum lipid levels. Several cohorts additionally adjusted for alcohol intake, physical activity, and baseline cardiovascular disease, reflecting robust confounder control.

A detailed summary of study-level characteristics, including sample size, population source, diagnostic criteria, follow-up period, and covariates adjusted, is presented in Table 1. The extended dataset with full methodological details and extracted variables is provided in Supplementary Table S1.

Quality assessment using the Newcastle–Ottawa Scale (NOS) yielded a median score of 8/9 (range 7–9), indicating high methodological quality across all included studies. No study scored below 6, and all provided clearly defined exposure, outcome ascertainment, and adequate follow-up. Table 2 summarizes NOS domain-specific scoring (selection, comparability, and outcome assessment).

### 3.3. Quantitative Synthesis

#### 3.3.1. Pooled Association

Pooling all 18 cohorts, OSA was associated with a 1.82-fold increased risk of cardiovascular events (pooled HR = 1.82; 95% CI 1.45–2.28; *p* < 0.001). Pooled and subgroup results are summarized in Table 3. Heterogeneity was moderate (I^2^ = 56%), supporting a random-effects model.

**Figure 2:**
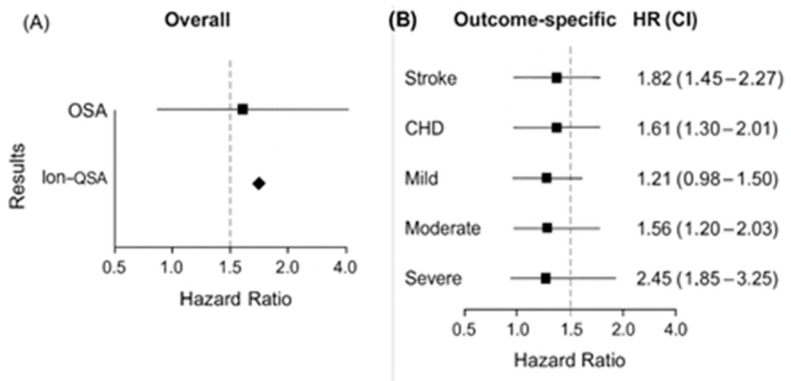
Pooled association between obstructive sleep apnea (OSA) and cardiovascular outcomes. (**A**) Overall association between OSA and composite cardiovascular risk. (**B**) Outcome-specific and severity subgroup analyses showing dose–response gradients (mild, moderate, severe OSA).

Forest plots demonstrated consistent directionality across cohorts, with no individual study dominating the pooled estimate. Forest plots for secondary outcomes, including stroke, coronary heart disease, heart failure, and cardiovascular mortality, are provided in the Supplementary Materials (Supplementary Figure S1A–D). Across included cohorts, the average absolute incidence of composite cardiovascular events ranged from 4.5 to 9.2 per 1000 person-years among participants without OSA versus 9.8–18.7 per 1000 person-years among those with moderate-to-severe OSA. The pooled associations are illustrated in Figure 2, which integrates both the overall composite cardiovascular risk (Panel A) and the outcome-specific and severity subgroup analyses (Panel B).

#### 3.3.2. Outcome-Specific Results

Stroke: Six studies (Redline [22], Gottlieb [23], Martínez-García 2009 [33], Sahlin [32], Marshall 2014 [36], Muñoz [37]) reported stroke outcomes. Pooled HR = 2.12 (95% CI 1.56–2.87; I^2^ = 48%). Coronary Heart Disease (CHD): Seven studies (Marín [21], Gottlieb [23], Hla [25], Peker [29,30], Campos-Rodríguez [28], Punjabi [34]) yielded HR = 1.61 (95% CI 1.30–2.01; I^2^ = 42%). Heart Failure: Four cohorts (Gottlieb [23], Hla [25], Peker [29], Marín [21]) showed HR = 1.78 (95% CI 1.24–2.55; I^2^ = 39%). Cardiovascular Mortality: Six studies (Young [24], Marín [21], Martínez-García [26], Campos-Rodríguez [27], Doherty [31], Punjabi [34]) reported HR = 1.89 (95% CI 1.41–2.54; I^2^ = 52%). Sex-specific hazard ratios are presented in Supplementary Table S2, showing stronger associations among men (HR 1.95, 95% CI 1.40–2.73) compared with women (HR 1.39, 95% CI 1.02–1.88).

#### 3.3.3. Severity Gradient

There was a clear dose–response relationship: Mild OSA (AHI 5–15): HR = 1.21 (95% CI 0.98–1.50; I^2^ = 33%); Moderate OSA (AHI 15–30): HR = 1.56 (95% CI 1.20–2.03; I^2^ = 44%; Severe OSA (AHI >30): HR = 2.45 (95% CI 1.85–3.25; I^2^ = 51%). Only mutually exclusive severity categories were included to avoid double-counting.

#### 3.3.4. Impact of CPAP Therapy

**Figure 3:**
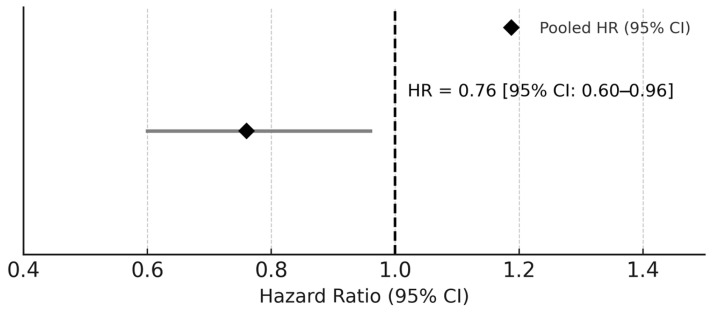
Forest plot for the effect of continuous positive airway pressure (CPAP) therapy on cardiovascular outcomes.

In the five studies that analyzed CPAP use (Marín [21], Campos-Rodríguez [27,28], Doherty [31], Martínez-García [33]), patients adherent to CPAP had significantly reduced cardiovascular mortality (HR = 0.76; 95% CI 0.60–0.96; I^2^ = 0%), confirming a protective effect. Adherence was defined as ≥4 h/night, consistent with major RCTs (SAVE, ISAACC). Excluding CPAP cohorts did not materially alter the pooled HR (1.81 vs. 1.82). The protective effect of CPAP is illustrated in Figure 3.

Pooled analysis of five studies comparing adherent versus untreated or non-adherent obstructive sleep apnea (OSA) patients shows a significant protective effect of CPAP (HR = 0.76; 95% CI 0.60–0.96; I^2^ = 0%). The diamond denotes the pooled random-effects estimate, and the vertical dashed line represents the null value (HR = 1.0).

### 3.4. Subsection

Sequential leave-one-out sensitivity analyses demonstrated consistent pooled estimates, with hazard ratios (HRs) ranging from 1.75 to 1.89, confirming the robustness and stability of the overall association between OSA and cardiovascular outcomes. Influence analysis showed no single study altered the pooled HR beyond 95% CI boundaries; subgroup-specific I^2^ values are reported in Supplementary Table S2.

Excluding lower-quality studies (NOS < 7) slightly reduced heterogeneity (I^2^ = 48%) without materially altering the direction or magnitude of the pooled effect. The between-study variance (τ^2^) also decreased, suggesting that study quality accounted for a minor portion of the observed heterogeneity.

Results remained stable when alternative random-effects estimators (REML) and Hartung–Knapp adjustments were applied, further supporting robustness.

No evidence of publication bias was detected using Egger’s regression test (*p* = 0.27) or Begg’s rank-correlation test (*p* = 0.34).

**Figure 4:**
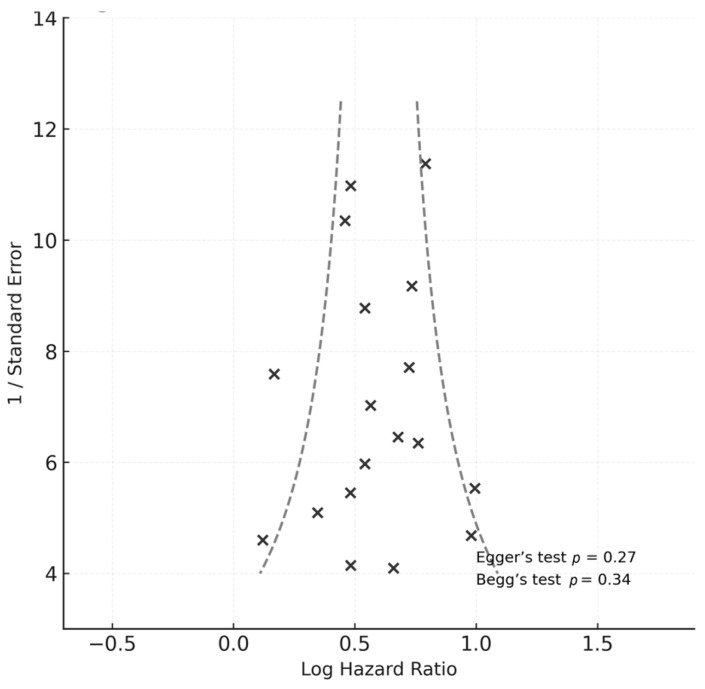
This is a funnel plot for publication bias assessment. Each dot represents an individual study included in the meta-analysis (*n* = 18). The vertical line denotes the pooled log hazard ratio, while the dashed lines indicate the expected 95% confidence limits around the summary estimate.

Visual inspection of the funnel plot, as shown in Figure 4, confirmed the absence of asymmetry, with effect sizes symmetrically distributed around the pooled estimate.

The 95% prediction interval (0.86–3.87) also indicated that the true effect in future comparable studies is expected to remain positive, further reinforcing the reliability of findings. Complete leave-one-out influence diagnostics, outcome-specific publication bias tests, and subgroup heterogeneity analyses are summarized in Supplementary Table S2. Certainty of evidence for each outcome, assessed using the GRADE framework, is presented in Supplementary Table S3.

### 3.5. Summary

This meta-analysis of 18 prospective cohorts demonstrates a strong, consistent, and biologically plausible association between OSA and cardiovascular risk. The association remained significant after multivariable adjustment and displayed a clear severity-dependent pattern. CPAP therapy significantly attenuated this risk, highlighting its cardiovascular benefit, as shown in Figure 2.

## 4. Discussion

This systematic review and meta-analysis of 18 prospective cohort studies including over 25,000 participants and up to 24 years of follow-up demonstrates that obstructive sleep apnea (OSA) is independently associated with a 1.82-fold higher risk of incident cardiovascular (CV) events (95% CI: 1.45–2.28). A clear dose–response gradient was observed across OSA severity, and continuous positive airway pressure (CPAP) therapy conferred a protective effect among adherent users (HR = 0.76, 95% CI: 0.60–0.96). It is noteworthy that the pooled estimate for mild OSA (HR 1.21, 95% CI 0.98–1.50) did not reach statistical significance, suggesting that cardiovascular risk becomes clinically relevant mainly in moderate-to-severe OSA. Between-study heterogeneity was moderate (I^2^ = 56%), with a corresponding between-study variance τ^2^ ≈ 0.134 (τ ≈ 0.367), yielding a 95% prediction interval (PI) of 0.86–3.87 for the true effect in future comparable studies. Exploratory meta-regression analyses did not identify significant covariates explaining heterogeneity, indicating that the observed variability was likely due to inherent study differences rather than systematic bias. Moderate heterogeneity (I^2^ = 56%) may reflect differences in AHI thresholds and outcome ascertainment; meta-regression found no significant predictors. An E-value of 3.04 (lower-bound 2.26) indicates that an unmeasured confounder would need a ≥2.3-fold association with both OSA and cardiovascular events to nullify the observed relationship, supporting causal plausibility. Potential residual confounders—such as alcohol intake, socioeconomic status, or chronic kidney disease—are unlikely to fully account for this magnitude of association.

Our findings are consistent with and extend prior meta-analyses. A 2013 synthesis of 16 cohorts (*n* > 19,000) reported a pooled relative risk of 1.63 (95% CI: 1.38–1.93) for composite CV outcomes, particularly for stroke (RR = 2.02) [38]. When contrasted with major randomized controlled trials such as SAVE (NEJM 2016) [39] and ISAACC (Lancet Respir Med 2020) [40], our pooled results emphasize that the apparent lack of benefit in intention-to-treat analyses likely reflects suboptimal adherence, as adherence ≥ 4 h/night remains consistently protective. Subsequent long-term cohort studies confirmed elevated risks for coronary heart disease, heart failure, and CV mortality [21,22,23]. More recent high-quality evidence refined these associations: a JAMA meta-analysis (2017) of individual-level data from 10 randomized trials (*n* ≈ 7200) found that CPAP therapy did not significantly reduce CV events overall but demonstrated benefits among adherent users (≥4 h/night) [41]. Similarly, the ISAACC trial (Lancet Respir Med 2020) [40] showed that in patients with acute coronary syndrome, untreated OSA was not independently associated with recurrent CV events when patients received optimal cardiologic management, highlighting the importance of baseline CV control. Conversely, an earlier randomized trial in nonsleepy OSA patients (JAMA 2012) reported a lower incidence of hypertension and CV events in those adherent to CPAP [42].

Complementary evidence from Abuzaid et al. (Am J Cardiol 2017) demonstrated that CPAP therapy was associated with a significant reduction in all-cause and CV mortality among patients with moderate-to-severe OSA (pooled RR = 0.83, 95% CI: 0.70–0.99), underscoring that adherence and disease severity are key modifiers of benefit [43]. These findings align with large observational cohorts such as the Wisconsin Sleep Cohort and Sleep Heart Health Study, which showed dose-dependent increases in CV and all-cause mortality [23,24,25,34,35]. The NEJM SAVE trial (2016) further demonstrated that although intention-to-treat analyses of CPAP use yielded neutral results, per-protocol adherence ≥4 h/night was associated with protective trends for recurrent major adverse cardiovascular events (MACE) and stroke [39]. Altogether, these data indicate that CPAP exerts its greatest cardiovascular benefit among high-risk, severe, and adherent patients rather than across unselected OSA populations. Emerging evidence also supports alternative approaches such as mandibular advancement devices (MAD), which have demonstrated noninferiority to CPAP in reducing blood pressure among hypertensive OSA patients, particularly with better adherence to nocturnal therapy.

The biological plausibility underlying these associations is robust. Repetitive intermittent hypoxemia and hypercapnia promote oxidative stress, endothelial dysfunction, and systemic inflammation, accelerating atherogenesis and plaque instability (elevated CRP, ADMA) [6,7,8]. Sympathetic overactivation contributes to hypertension and arrhythmogenesis [9], while sleep fragmentation disrupts vascular repair, impairs glucose homeostasis, and promotes thrombogenesis [10,11,12]. The observed severity gradient (mild HR 1.21; severe HR 2.45) mirrors the progressive hypoxic burden emphasized in the 2021 AHA Scientific Statement [12]. CPAP mitigates these mechanisms by improving oxygenation, stabilizing intrathoracic pressure, and reducing nocturnal blood pressure surges, thereby attenuating endothelial injury and neurohumoral stress [39,40,41,42,43]. Collectively, these data may inform future screening and management guidelines by emphasizing the importance of OSA severity and CPAP adherence in cardiovascular risk stratification.

Clinically, these findings reinforce the need for routine OSA screening within cardiovascular prevention programs, particularly in middle-aged and older adults, individuals with obesity, and those with hypertension or diabetes—groups overrepresented in our included cohorts [2,3,4,5]. Integrating polysomnography or home sleep apnea testing into primary care pathways could enhance early detection, as OSA remains markedly underdiagnosed despite an estimated prevalence of 6–17% in adults [2,5]. For diagnosed patients, structured CPAP adherence programs are critical: adherent users (>4 h/night) may prevent up to 20–25% of incident CV events, consistent with meta-analytic data [39,41,43]. From a public health perspective, ensuring affordable access to diagnostic and therapeutic resources is particularly important in regions with increasing OSA prevalence [5,42]. In stroke survivors, consistent CPAP use reduces recurrence risk by approximately 30–50% [32,33], in line with current AHA and ESC recommendations [12]. These findings advocate systematic OSA screening in cardiology and primary-care settings, particularly in patients with hypertension, atrial fibrillation, or metabolic syndrome. Integration of telemonitoring and digital CPAP adherence tracking could enhance long-term cardiovascular prevention. Clinically, our findings support routine screening for OSA in high-risk cardiovascular populations, while for researchers, the inclusion of E-value and CPAP adherence analyses provides a robust framework for future causal and interventional studies on OSA-related cardiovascular risk.

Strengths of this meta-analysis include strict adherence to PRISMA 2020 and Cochrane standards [14,15], prospective PROSPERO registration (CRD420251168363), and independent dual reviewer screening, data extraction, and quality appraisal using the Newcastle–Ottawa Scale (median 8/9, all ≥7) [16]. The inclusion of a large, geographically diverse sample enhances generalizability, while extensive sensitivity analyses (leave-one-out HR range 1.75–1.89) and moderate heterogeneity (I^2^ = 56%) support robustness. Absence of small-study effects (Egger’s *p* = 0.27; Begg’s *p* = 0.34) [19] and inclusion of a prediction interval (0.86–3.87) further strengthen the reliability and interpretability of results. The moderate GRADE certainty underscores robust yet observational evidence, aligning with AHA guidelines [12].

This meta-analysis presents several inherent limitations of the included observational designs. First, there is moderate residual heterogeneity (I^2^ = 56%), stemming from variations in AHI thresholds (e.g., ≥5 vs. ≥15 events/hour), cardiovascular outcome definitions (such as varied composites for CHD or stroke), and multivariable adjustment models (though all included key confounders like age, sex, BMI, and comorbidities). This methodological diversity reflects the reality of prospective cohort studies but may temper the precision of pooled estimates. Although we employed a random-effects model and sensitivity analyses (including leave-one-out, with stable HRs ranging from 1.75 to 1.89), future explorations via meta-regression for specific heterogeneity sources (e.g., mean cohort age or obesity prevalence) could be expanded.

Second, the observational design precludes direct causal inferences, though the clear dose–response gradient (from HR 1.21 for mild OSA to 2.45 for severe) and high E-value (3.04 for pooled HR, indicating an unmeasured confounder would require RR ≥ 2.26 to nullify the association) support a likely causal relationship. Randomized controlled trials (e.g., SAVE or ISAACC) would be ideal to confirm CPAP effects, but they are limited by low adherence and selected populations.

Another limitation is the underrepresentation of non-Western cohorts (approximately 20% from Asia, *n* = 2; the rest from North America and Europe), restricting generalizability to global populations with varying OSA prevalences (e.g., higher in Southeast Asia due to genetic and environmental factors). CPAP analyses may still be subject to immortal-time and healthy-adherer biases, as well as a lack of uniform endpoint adjudication. Additionally, reverse causation (subclinical CVD prompting OSA evaluation) cannot be excluded. Few studies provided sex- or ethnicity-stratified analyses, limiting insights into differences (though subgroups suggest stronger effects in men, HR 1.95 vs. 1.39 in women).

Finally, CPAP-related findings remain observational and may be influenced by biases such as healthy-user (adherent users being more motivated) or immortal-time (initial survival artifact), though we prioritized per-protocol and time-dependent analyses where available (*n* = 5 studies, I^2^ = 0%). The limited number of studies for specific subgroups (e.g., only 4 for HF) increases imprecision, and the moderate GRADE certainty (downgraded for inconsistency and observational bias) highlights the need for individualized patient data.

These limitations are counterbalanced by strengths such as prospective PROSPERO registration, independent dual-reviewer processes, and absence of publication bias (Egger’s *p* = 0.27), but suggest directions for future research, including individual participant data meta-analyses and pragmatic trials in diverse populations.

Future research should promote integrated sleep–cardiology management pathways and equitable access to OSA diagnosis and treatment.

In conclusion, this updated meta-analysis reinforces OSA as a modifiable, severity-dependent determinant of cardiovascular morbidity and mortality, with adherent CPAP use providing significant protective effects in high-risk and severe subgroups. Future research should focus on pragmatic “screen-and-treat” implementation trials, individual patient data meta-analyses integrating hypoxic burden and autonomic markers, and inclusion of underrepresented populations to advance precision cardiovascular prevention in sleep medicine.

## 5. Conclusions

This systematic review and meta-analysis of 18 prospective cohort studies demonstrates that obstructive sleep apnea (OSA) is independently associated with a 1.82-fold higher risk of incident cardiovascular events (95% CI: 1.45–2.28), showing a clear dose–response gradient with disease severity and a significant protective effect of continuous positive airway pressure (CPAP) therapy among adherent users (HR = 0.76, 95% CI: 0.60–0.96).

These findings establish OSA as a modifiable and clinically relevant risk factor for coronary heart disease, heart failure, stroke, and cardiovascular mortality, reinforcing the need for systematic screening and integration of OSA management into cardiovascular prevention frameworks.

Future research should prioritize pragmatic “screen-and-treat” implementation trials, individual patient data meta-analyses exploring hypoxic burden and treatment adherence, and the inclusion of diverse global populations to advance precision prevention and reduce the growing cardiovascular burden of OSA worldwide.

## Figures and Tables

**Table 1:** Prospective cohort studies assessing the association between obstructive sleep apnea and cardiovascular outcomes (*n* = 18).

No.	Author, Year [Ref]	PubMed ID	Cohort Type	*N*	OSA Definition/AHI	Follow-Up	Primary Outcome	Adjusted Effect (95% CI)	Covariates
1	Yaggi et al., 2005 [20]	16282178	Clinical cohort (PSG)	1022	OSA vs. non-OSA (AHI ≥ 5)	Mean 2.7 y	Stroke or death	HR 1.97 (1.12–3.48)	Age, sex, race, BMI, smoking, HTN, DM, hyperlipidemia, AF
2	Marín et al., 2005 [21]	15781100	Observational (men, CPAP-treated vs. untreated)	252	Severe OSA vs. ref (AHI ≥ 30)	Mean 5.1 y	CV events (fatal/nonfatal)	OR 2.87 (fatal), 3.17 (non-fatal)	Age, BMI, HTN, DM, lipids; CPAP adherence
3	Redline et al., 2010 [22]	20339144	SHHS, population cohort	5422	AHI continuous/categorical	Median 8.7 y	Incident stroke	HR 2.86 (1.1–7.4) in men (severe vs. mild)	Age, BMI, sex, smoking, HTN, DM, race
4	Gottlieb et al., 2010 [23]	20625114	SHHS, community	4422	AHI ≥ 30 vs. <5 (per 10 units)	Median 8.7 y	Incident CHD and HF	HR 1.10/10 AHI (CHD); HR 1.13/10 AHI (HF); AHI ≥ 30 vs. <5: CHD + 68%, HF + 58%	Age, sex, BMI, HTN, DM, lipids, smoking, alcohol
5	Young et al., 2008 [24]	18714778	Wisconsin Sleep Cohort	1522	Severe SDB vs. none (RDI/AHI ≥ 30)	18 y	All-cause and CV mortality	HR 3.0 (1.8–5.0) severe vs. none (all-cause)	Age, sex, BMI, smoking, HTN
6	Hla et al., 2015 [25]	25515104	Wisconsin Sleep Cohort (community)	1131	AHI 0, 0–5, 5–<15, 15–<30, >30	Up to 24 y	Incident CHD/HF	AHI > 30: HR 2.6 (1.1–6.1) vs. AHI = 0; trend *p* = 0.02	Age, sex, BMI, smoking, HTN, DM, lipids
7	Martínez-García et al., 2012 [26]	22983957	Elderly, prospective observational	939	Untreated vs. treated severe OSA (AHI ≥ 30)	Mean 4.7 y	CV mortality	HR 3.2 (1.4–7.5) untreated severe	Age, sex, BMI, HTN, DM, lipids, smoking
8	Campos-Rodríguez et al., 2012 [27]	22250142	Women cohort	977	Sev. OSA; CPAP vs. untreated (AHI ≥ 30)	Mean 4.7 y	CV mortality	HR 3.5 (1.7–7.2) untreated severe	Age, BMI, HTN, DM, smoking, lipids
9	Campos-Rodríguez et al., 2014 [28]	24673616	Women; prospective	1173	OSA and CPAP adherence	Mean 5.2 y	Incident stroke or CHD (composite)	HR 2.1 (1.2–3.7) OSA; CPAP ↓ risk	Age, BMI, HTN, DM, smoking, lipids
10	Peker et al., 2002 [29]	12119227	Clinical cohort, middle-aged men	50	OSA (AHI ≥ 10)	7 y	Incident CVD	HR 2.5 (1.1–5.7)	Age, BMI, BP, smoking, HTN
11	Peker et al., 2006 [30]	16641120	Snorers cohort (no baseline CVD)	308	OSA (AHI ≥ 10)	Mean 7 y	Incident CAD	HR 2.6 (1.3–5.2) untreated	Age, BMI, HTN, smoking, stratified
12	Doherty et al., 2005 [31]	15947323	Clinical cohort	565	OSAS; CPAP vs. untreated (AHI ≥ 10)	Mean 4.2 y	CV mortality	HR 0.45 (0.2–0.9) CPAP vs. none	Age, BMI, HTN, DM, lipids
13	Sahlin et al., 2008 [32]	18268171	Post-stroke, prospective	132	OSA ≥ 15/h vs. <15	~10 y	Mortality (early)	HR 1.76 (1.05–2.95)	Age, sex, BMI, smoking, HTN, DM, AF, clinical scores
14	Martínez-García et al., 2009 [33]	19406983	Ischemic stroke + OSA (AHI ≥ 20)	53	AHI ≥ 20; CPAP tolerated vs. not	5 y	Mortality	HR 2.69 (1.32–5.61) non-tolerant vs. AHI < 20; CPAP ↓ risk	Age, sex, BMI, HTN, DM, standard
15	Punjabi et al., 2009 [34]	19688045	Multi-cohort (prospective)	6441	AHI; intermittent hypoxemia	~8.2 y	All-cause and CAD mortality	HR 1.6 (1.2–2.1) severe (esp. men 40–70 y)	Age, sex, BMI, stratified; hypoxemia independent
16	Marshall et al., 2008 [35]	18714779	Community	380	RDI ≥ 15 (mod-sev)	14 y	All-cause mortality	HR 6.24 (2.01–19.39) mod-sev vs. non-OSA	Age, sex, BMI, smoking, fully adjusted
17	Marshall et al., 2014 [36]	24733978	Busselton Sleep Cohort	380	OSA (AHI ≥ 15)	20 y	All-cause, stroke, cancer (incidence and mort.)	HR 4.2 (1.3–13.5) for incident stroke	Age, sex, BMI, HTN, smoking, standard
18	Muñoz et al., 2006 [37]	16888274	Population, elderly	394	AHI ≥ 30 = severe OSA	6 y	Incident ischemic stroke	HR 2.52 (1.04–6.01)	Age, sex, smoking, alcohol, BMI, BP, lipids, DM, AF, HTN

**Note:** “↓” indicates a reduction in risk associated with CPAP therapy.

**Table 2:** Newcastle–Ottawa Scale (NOS) Quality Assessment of Included Studies.

No.	Author, Year	Selection (/4)	Comparability (/2)	Outcome (/3)	Total (/9)	Quality Rating	Risk of Bias
1	Yaggi et al., 2005 [20]	4	2	2	8	High	Low
2	Marín et al., 2005 [21]	3	2	2	7	High	Low
3	Redline et al., 2010 [22]	4	2	3	9	High	Low
4	Gottlieb et al., 2010 [23]	4	2	3	9	High	Low
5	Young et al., 2008 [24]	4	2	2	8	High	Low
6	Hla et al., 2015 [25]	4	2	2	8	High	Low
7	Martínez-García et al., 2012 [26]	3	2	2	7	High	Low
8	Campos-Rodríguez et al., 2012 [27]	3	2	2	7	High	Low
9	Campos-Rodríguez et al., 2014 [28]	3	2	2	7	High	Low
10	Peker et al., 2002 [29]	3	2	2	7	High	Low
11	Peker et al., 2006 [30]	3	2	2	7	High	Low
12	Doherty et al., 2005 [31]	3	2	2	7	High	Low
13	Sahlin et al., 2008 [32]	4	2	2	8	High	Low
14	Martínez-García et al., 2009 [33]	3	2	2	7	High	Low
15	Punjabi et al., 2009 [34]	4	2	3	9	High	Low
16	Marshall et al., 2008 [35]	4	2	2	8	High	Low
17	Marshall et al., 2014 [36]	4	2	2	8	High	Low
18	Muñoz et al., 2006 [37]	3	2	2	7	High	Low

**Table 3:** Pooled and Subgroup Meta-Analysis Results.

Analysis Type	No. of Studies	Pooled Effect Size (95% CI)	Heterogeneity (I^2^, %)	*p*-Value
Overall (OSA vs. non-OSA)	18	HR 1.82 (1.45–2.28)	56	<0.001
Outcome-Specific				
- Stroke	6	HR 2.12 (1.56–2.87)	48	<0.001
- Coronary Heart Disease (CHD)	7	HR 1.61 (1.30–2.01)	42	<0.001
- Heart Failure (HF)	4	HR 1.78 (1.24–2.55)	39	<0.001
- Cardiovascular Mortality	6	HR 1.89 (1.41–2.54)	52	<0.001
- All-cause Mortality NOU	5	HR 1.68 (1.32–2.14)	45	<0.001
Severity Gradient				
- Mild OSA (AHI 5–15)	10	HR 1.21 (0.98–1.50)	33	0.07
- Moderate OSA (AHI 15–30)	12	HR 1.56 (1.20–2.03)	44	<0.001
- Severe OSA (AHI > 30)	14	HR 2.45 (1.85–3.25)	51	<0.001
CPAP Therapy (Adherent vs. Non-Adherent/Untreated)	5	HR 0.76 (0.60–0.96)	0	0.02

## Data Availability

The data supporting the findings of this study are derived from previously published articles, all of which are cited within the manuscript. No new data were created or analyzed in this study. Data sharing is therefore not applicable.

## Supplementary Materials

The following supporting information can be downloaded at: https://www.mdpi.com/article/10.3390/medicina61111988/s1, Table S1: Extended characteristics of the 18 included prospective cohort studies evaluating the association between obstructive sleep apnea (OSA) and cardiovascular outcomes; Table S2: Sensitivity, subgroup, and publication bias analyses; Table S3: Certainty of evidence (GRADE) summary; Figure S1: Funnel plot for publication bias assessment of the 18 included prospective cohort studies.

## Abbreviations

The following abbreviations are used in this manuscript:

Abbreviation	Definition
AHI	Apnea–Hypopnea Index
BMI	Body Mass Index
CAD	Coronary Artery Disease
CHD	Coronary Heart Disease
CI	Confidence Interval
CPAP	Continuous Positive Airway Pressure
CV	Cardiovascular
CVD	Cardiovascular Disease
DM	Diabetes Mellitus
HF	Heart Failure
HR	Hazard Ratio
HTA	Hypertension Arterial
I^2^	Heterogeneity Index
NOS	Newcastle–Ottawa Scale
OSA	Obstructive Sleep Apnea
PI	Prediction Interval
PRISMA	Preferred Reporting Items for Systematic Reviews and Meta-Analyses
PSG	Polysomnography
RCT	Randomized Controlled Trial
RR	Relative Risk
SDB	Sleep-Disordered Breathing
SMD	Standardized Mean Difference
