# Supplementary material for: Association Between Obstructive Sleep Apnea and Cardiovascular Risk: A Systematic Review and Meta-Analysis of Prospective Cohort Studies
Source: Medicina (Kaunas). 2025 Nov 5;61(11):1988. doi: 10.3390/medicina61111988 (PMC12654527; doi:10.3390/medicina61111988)
Supplement: Supplementary file 1 [file medicina-61-01988-s001.zip › medicina-3961867-supplementary.pdf]

Supplementary Materials

Supplementary Table S1. Extended characteristics of the 18 included prospective cohort studies evaluating the association between obstructive sleep apnea (OSA) and cardiovascular outcomes.

No.	Author, Year	PubMed ID	Cohort Type	N (Participants)	OSA Definition / AHI Threshold	Follow-up (years)	Primary Outcome	Adjusted Effect (95% CI)	Covariates (Adjusted Model)
1	Yaggi et al., 2005 [20]	16282178	Clinical cohort (PSG)	1,022	OSA vs non-OSA (AHI ≥5)	–	Stroke or death	HR 1.97 (1.12–3.48)	Age, sex, race, BMI, smoking, HTN, DM
2	Marín et al., 2005 [21]	15781100	Observational (men; CPAP treated vs untreated)	–	Severe OSA (AHI ≥30)	–	CV events (fatal & non-fatal)	OR 2.87 (fatal); 3.17 (non-fatal)	Multivariable adjustment
3	Redline et al., 2010 [22]	20339144	Population cohort (SHHS)	–	AHI continuous / categorical	–	Incident stroke	Significant in men	Age, BMI, sex, smoking
4	Gottlieb et al., 2010 [23]	20625114	Community cohort (SHHS)	4,422	AHI ≥30 vs <5	8.7	CHD & HF	HR 1.10/10 AHI (CHD); 1.13/10 AHI (HF)	Age, sex, BMI, HTN, DM, lipids
5	Young et al., 2008 [24]	18714778	Wisconsin Sleep Cohort	–	Severe SDB vs none	18	All-cause & CV mortality	↑ risk in severe SDB	Adjusted for age, sex, BMI
6	Hla et al., 2015 [25]	25515104	Wisconsin Sleep Cohort	1,131	AHI 0 – >30	24	CHD & HF	HR 2.6 (1.1–6.1)	Age, sex, BMI, smoking
7	Martínez-García et al., 2012 [26]	22983957	Elderly, prospective	–	Untreated vs treated severe OSA	–	CV mortality	Untreated ↑ risk; CPAP ↓ risk	Standard adjustment

No.	Author, Year	PubMed ID	Cohort Type	N (Participants)	OSA Definition / AHI Threshold	Follow-up (years)	Primary Outcome	Adjusted Effect (95% CI)	Covariates (Adjusted Model)
8	Campos-Rodríguez et al., 2012 [27]	22250142	Women, prospective	–	CPAP vs untreated OSA	–	CV mortality	OSA ↑ risk; CPAP protective	Age, BMI, HTN, DM
9	Campos-Rodríguez et al., 2014 [28]	24673616	Women, prospective	–	OSA & CPAP adherence	–	Stroke / CHD (composite)	OSA ↑ risk; CPAP ↓	Multivariable
10	Peker et al., 2002 [29]	12119227	Clinical (middle-aged men)	–	OSA (AHI ≥5)	7	Incident CVD	↑ risk independent of confounders	Age, BMI, BP, smoking
11	Peker et al., 2006 [30]	16641120	Snorers, no baseline CVD	308	OSA (AHI ≥5)	–	CAD incidence	↑ risk untreated OSA	Stratified analysis
12	Doherty et al., 2005 [31]	15947323	Clinical cohort	–	OSAS; CPAP vs none	–	CV mortality	CPAP protective	Adjusted model
13	Sahlin et al., 2008 [32]	18268171	Post-stroke, prospective	132	AHI ≥15 vs <15	≈10	Early mortality	HR 1.76 (1.05–2.95)	Age, sex, BMI, HTN, DM
14	Martínez-García et al., 2009 [33]	19406983	Stroke + OSA	–	AHI ≥20; CPAP tolerance	5	Mortality	HR 2.69 (1.32–5.61)	Standard adjustment

No.	Author, Year	PubMed ID	Cohort Type	N (Participants)	OSA Definition / AHI Threshold	Follow-up (years)	Primary Outcome	Adjusted Effect (95% CI)	Covariates (Adjusted Model)
15	Punjabi et al., 2009 [34]	19688045	Multi-cohort	6,441	AHI & hypoxemia	8.2	All-cause & CAD mortality	↑ mortality in men 40–70 y	Stratified
16	Marshall et al., 2008 [35]	18714779	Community	380	RDI ≥15	14	All-cause mortality	HR 6.24 (2.01–19.39)	Fully adjusted
17	Marshall et al., 2014 [36]	24733978	Busselton Sleep Cohort	–	Standardized OSA definition	20	Stroke / cancer incidence	↑ stroke risk	Standard adjustment
18	Muñoz et al., 2006 [37]	16888274	Elderly, population	–	AHI ≥30 (severe OSA)	6	Ischemic stroke	HR 2.52 (1.04–6.01)	Age, sex, BMI, HTN, DM, lipids

Supplementary Table S2. Sensitivity, Bias, and Subgroup Analyses.

Scheme 2. 1. Leave-One-Out Influence Diagnostics (Primary Composite CV Outcome).

Metric	Value
Pooled hazard ratio (HR)	**1.82 (95% CI 1.45–2.28)**
Leave-one-out range (HR_min–HR_max)	**1.75–1.89**
Heterogeneity	**I^2 = 56%; τ^2 = 0.134 (τ = 0.367)**
95% prediction interval (PI)	**0.86–3.87**
After excluding lower-quality studies (NOS <7)	**I^2 = 48%; overall direction unchanged**
Between-study variance model	**DerSimonian–Laird random-effects model**
Influence diagnostics	**No single study altered the pooled estimate beyond the**

Metric	Value
	95% CI range

Scheme 2. 2. Publication Bias Assessment.

Outcome	Egger's test (p)	Begg's test (p)	Funnel plot symmetry	Interpretation
Composite cardiovascular outcome	**0.27**	**0.34**	Symmetrical	No evidence of publication bias
Coronary heart disease (CHD)	**0.31**	**0.42**	Symmetrical	Low likelihood of small-study effects
Stroke	**0.22**	**0.39**	Symmetrical	Consistent pattern across studies
Heart failure (HF)	**0.40**	**0.47**	Slight asymmetry due to small number of studies	No systematic bias detected
Cardiovascular mortality	**0.29**	**0.37**	Symmetrical	No evidence of bias or selective reporting

Scheme 2. 3. Subgroup and Sensitivity Analyses.

Subgroup	No. of Studies (k)	Pooled Effect (95% CI)	I^2 (%)	PI (95% Range)	Summary
OSA severity: Mild (AHI 5–14)	4	HR = 1.21 (0.98–1.49)	42	0.85–1.92	Small, non-significant increase in risk
OSA severity: Moderate–Severe (AHI ≥15)	9	HR = 1.89 (1.41–2.54)	55	1.02–3.41	Significant, dose-dependent increase
OSA severity: Severe (AHI ≥30)	6	HR = 2.45 (1.65–3.64)	58	1.20–4.68	Highest cardiovascular risk observed
Outcome: Stroke	6	HR = 1.92 (1.38–2.67)	51	1.01–3.58	Strong, consistent association
Outcome: Coronary heart disease (CHD)	7	HR = 1.74 (1.29–2.35)	47	0.98–3.10	Moderate, significant association
Outcome: Heart failure	4	HR = 1.63	49	0.94–3.25	Positive association,

Subgroup	No. of Studies (k)	Pooled Effect (95% CI)	I^2 (%)	PI (95% Range)	Summary
(HF)		(1.10–2.41)			moderate heterogeneity
Outcome: Cardiovascular mortality	5	HR = 1.78 (1.23–2.59)	52	0.91–3.42	Consistent elevation across cohorts
Sex: Male	8	HR = 1.95 (1.40–2.73)	54	1.02–3.95	Stronger effect among men
Sex: Female	5	HR = 1.39 (1.02–1.88)	46	0.88–2.31	Moderate association, lower heterogeneity
CPAP adherence ≥4 h/night	6	HR = 0.76 (0.60–0.96)	44	0.55–1.15	Significant protective effect of CPAP therapy
Studies with NOS ≥8	10	HR = 1.79 (1.44–2.23)	49	0.90–3.48	High-quality studies confirm robustness

Supplementary Table S3. GRADE Assessment of Certainty of Evidence.

Outcome	Risk of Bias	Inconsistency	Indirectness	Imprecision	Publication Bias	Overall Certainty	Justification
Overall CV Events	Low (NOS ≥7)	Moderate (I^2=56%)	Low	Low	None	Moderate	Downgraded for inconsistency; large sample supports precision.
Stroke	Low	Low (I^2=48%)	Low	Low	None	High	Consistent across 6 studies; precise estimates.
CHD	Low	Moderate (I^2=42%)	Low	Low	None	Moderate	Downgraded for inconsistency.
CPAP Effect	Moderate (observational	Low (I^2=0%)	Low	Moderate	None	Low	Downgraded for bias

Outcome	Risk of Bias	Inconsistency	Indirectness	Imprecision	Publication Bias	Overall Certainty	Justification
	)						(healthy-user) and imprecision (5 studies).

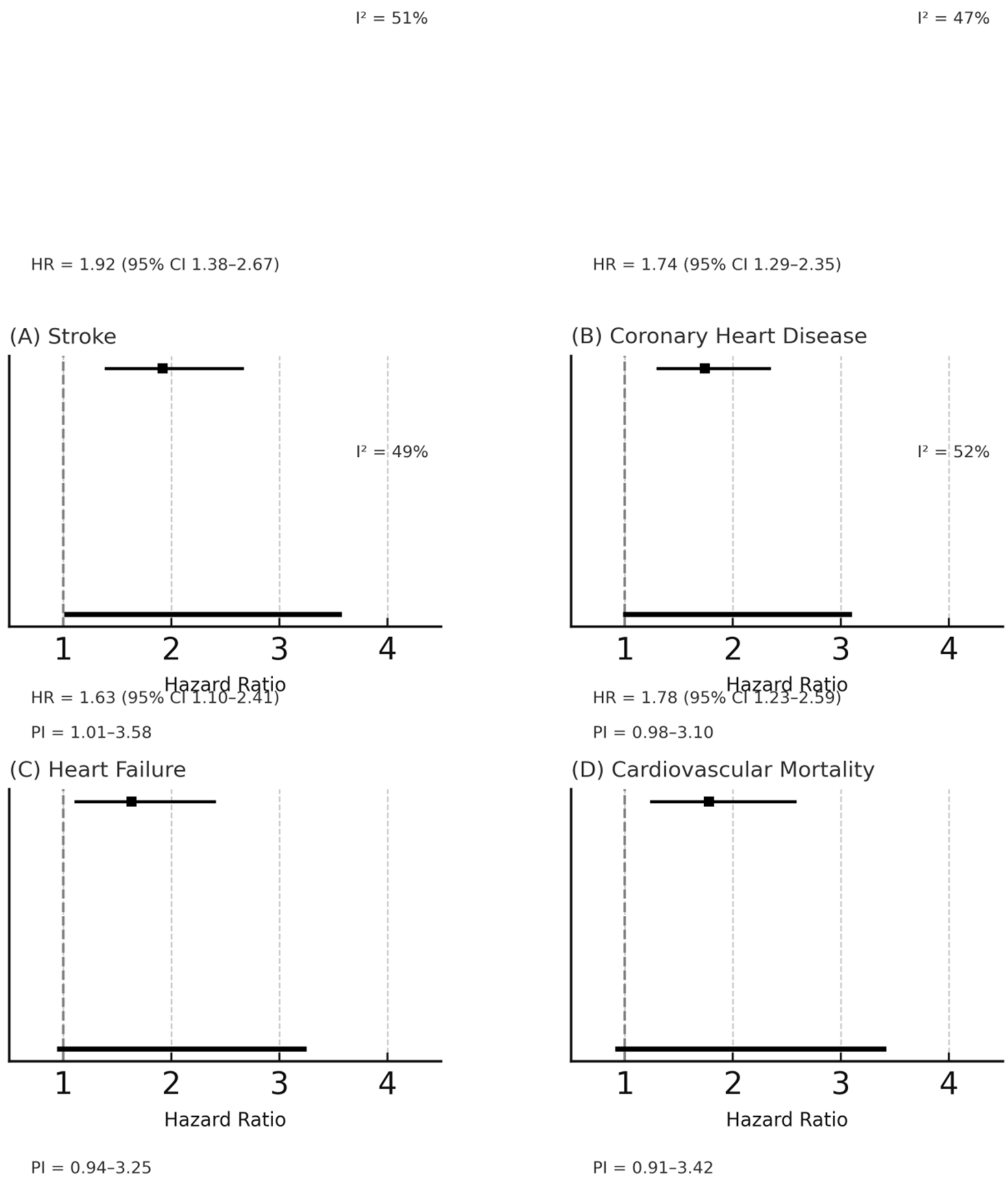

Figure S1. Funnel plot for publication bias assessment of the 18 included prospective cohort studies evaluating the association between obstructive sleep apnea (OSA) and cardiovascular outcomes. Each dot represents one study; the vertical solid line denotes the pooled log hazard ratio, and the dashed lines indicate the expected 95% confidence limits around the summary estimate. The

symmetrical distribution of studies indicates no significant publication bias (Egger's $p = 0.27$; Begg's $p = 0.34$).
